# Surgically-Induced Necrotizing Scleritis After Scleral Buckling With Stenotrophomonas maltophilia Infection

**DOI:** 10.7759/cureus.53876

**Published:** 2024-02-08

**Authors:** Yoshihiro Nakagawa, Takahiro Suzuki, Yasuyuki Suzuki

**Affiliations:** 1 Ophthalmology, Tokai University Hospital, Isehara, JPN

**Keywords:** scleral necrosis, postoperative complications, stenotrophomonas maltophilia infection, scleral buckle infection, surgically induced necrotizing scleritis

## Abstract

Surgically induced necrotizing scleritis (SINS) is a rare inflammatory disease of the sclera that occurs following ocular surgery, specifically pterygium surgery and scleral buckling. Here, we report a case of SINS in a 78-year-old female patient after segmental scleral buckling for rhegmatogenous retinal detachment. The retina was restored after scleral buckling, and the postoperative course was uneventful. However, the patient developed ocular discharge and conjunctival hyperemia, indicating infection, after two months. The sclera became thinner and intraocular inflammation developed after buckle removal. *Stenotrophomonas maltophilia* was isolated from the ocular discharge, and the patient was treated with antibacterial agents susceptible to the bacteria. However, her symptoms persisted, and corrected visual acuity decreased from 20/25 to 20/1000. Oral steroid treatment was initiated because of the suspicion of SINS. Intraocular inflammation gradually subsided, the thin sclera was covered by conjunctival tissue, and the patient’s corrected visual acuity improved to 20/32, which stabilized her condition. Infection with *Stenotrophomonas maltophilia* after scleral buckling is extremely rare, and SINS development in such cases is unprecedented.

## Introduction

Scleral buckling for rhegmatogenous retinal detachment is an effective and traditional surgical procedure that restores the retina in most cases. Postoperative complications include elevated intraocular pressure, vitreous or expulsive hemorrhage, and retinal redetachment. Additionally, infection should be first suspected in cases of severe inflammation. Scleral buckling, an external ophthalmic procedure, involves implanting a silicone sponge on the scleral surface, which causes site infection. The incidence of scleral buckle infections varies but is estimated to range from 1% to several percent, with gram-positive bacteria being the most common cause, followed by gram-negative bacilli [1,2]. Surgically induced necrotizing scleritis (SINS) is another rare disease that causes severe scleral inflammation after ophthalmic surgery and is believed to be autoimmune in origin. SINS, associated with infection, necessitates distinguishing between postoperative inflammation caused by infection and autoimmunity. Here, we present a case of postoperative scleral buckle infection caused by *Stenotrophomonas maltophilia* (Gram-negative rod), a rare etiologic agent. This case describes an uncommon occurrence of scleral thinning, considered SINS, following a scleral buckle infection.

## Case presentation

**Figure 1:**
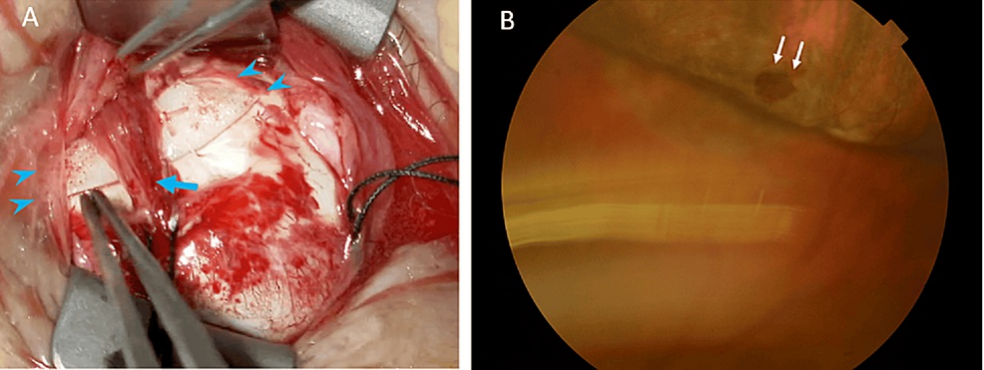
Segmental scleral buckling restored retina (left eye images) (A) A silicone sponge buckle (arrowheads) is inserted under the superior rectus muscle (arrow) and fixed. (B) The retinal tear (arrows) is closed by buckling.

A 78-year-old female patient visited her primary care ophthalmologist one week after noticing floaters in her left eye. The patient was referred to our hospital with a diagnosis of rhegmatogenous retinal detachment in the left eye. Her medical history included hypertension and bronchial asthma but no history of diabetes, autoimmune disease, or immunosuppressive conditions or medications. Regarding ocular history, there was a case of cataract surgery on the left eye several years earlier. The corrected visual acuity was 20/250 in the right eye and 20/25 in the left eye at the initial visit. She had a refractive error of -4D in the right eye and -1D in the left eye, and intraocular pressure of 21 mmHg in both eyes. The right eye demonstrated nuclear and posterior subcapsular cataracts and atrophic macular foci. The left eye, under mydriasis, exhibited temporal superior low-height retinal detachment within one quadrant with a single retinal tear. No vitreous opacity was observed, nor were there any degenerative lesions other than in the area of retinal detachment. A segmental scleral buckling was performed under retrobulbar anesthesia five days after the initial visit. Briefly describing the surgery, the superior temporal conjunctiva was incised, traction threads were passed through the superior and external rectus muscles, and cryocoagulation was performed from the sclera to the retinal tear. A silicone sponge (MIRA Inc. #506) was then placed under the superior rectus muscle, sutured and fixed by 5-0 polyester. The retina was restored (Figure 1) and no significant inflammation was observed postoperatively. 0.1% betamethasone and 0.5% moxifloxacin eye drops were used four times a day and the patient continued to be examined every few weeks without any special ocular symptoms.

**Figure 2:**
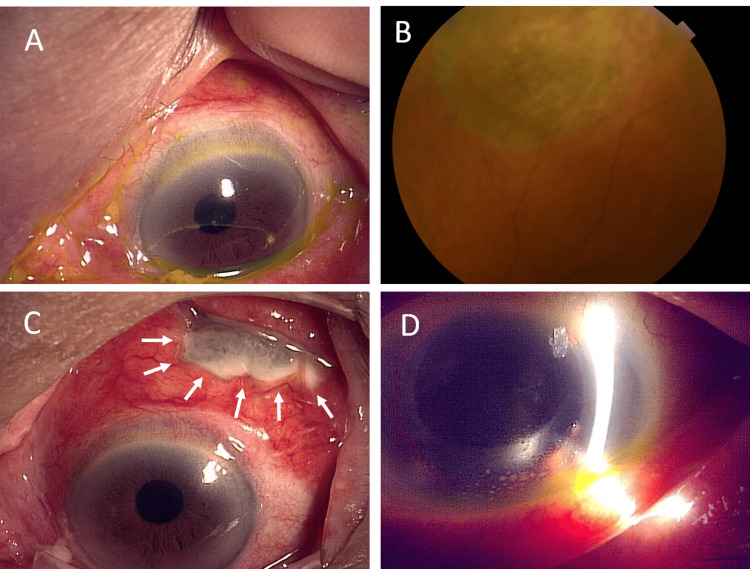
Postoperative inflammation (all left eye images) (A) Two months after the initial surgery, the upper conjunctival hyperemia and ocular discharge demonstrated an ocular surface stained with fluorescein), but with no buckle exposure on the conjunctiva. (B) Fundus image 10 days after buckle removal; there is no evidence of retinal redetachment, but the area of previous detachment has atrophic changes. (C) Three months after the initial surgery; the sclera in the area where the buckle was removed is necrotic (arrows). (D) Multiple muton fat-like keratic precipitates are seen on the posterior cornea.

However, after two months, the patient complained of ocular pain, increased conjunctival hyperemia, and ocular discharge in the left eye (Figure 2A). There were no signs of inflammation in the anterior segment and no changes in the reattached retina. The buckle suspected of being infected was removed on postoperative day 77 revealing *S. maltophilia* in ocular discharge. Based on the results of drug sensitivity testing, levofloxacin eye drops, which are susceptible, were continued six times a day, then the ocular pain improved and discharge was diminished. Bacterial culture results of the removed buckle were negative, and retinal redetachment did not occur (Figure 2B). One month after buckle removal, scleral thinning occurred rapidly in the area where the buckle was removed, with ocular pain in her left eye (Figure 2C). Retinal restoration was maintained, but inflammatory cells and multiple mutton fat-like keratic precipitates appeared in the anterior chamber (Figure 2D). Another month later, corrected visual acuity in the left eye decreased to 20/1000 because of inflammation of the anterior chamber and vitreous opacity. Bacterial culture and comprehensive polymerase chain reaction testing of the anterior chamber fluid were undetectable both bacteria and virus. Therefore, based on the findings of scleral necrosis consistent with the site of buckle removal and intense intraocular inflammation without organisms, SINS was suspected, and oral steroids were initiated.

**Figure 3:**
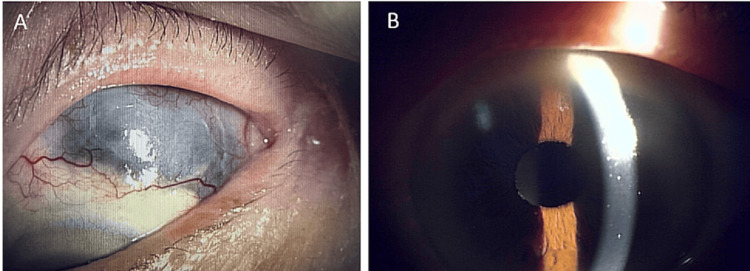
After anti-inflammatory therapy for SINS (left eye images) (A) The choroid in the scleral necrosis area is covered with conjunctival tissue and appears grayish-white two years after scleral buckling. (B) Anterior segment inflammation has disappeared two years after scleral buckling. SINS: Surgically induced necrotizing scleritis

Oral methylprednisolone (30 mg/day) resolved intraocular inflammation, covering the thin sclera with conjunctival tissue (Figure 3). The corrected visual acuity improved to 20/32 with tapering the dosage of steroids every few weeks. At two years postoperatively, the inflammation has remained unnoticeable with low-dose (1 mg/day) methylprednisolone.

## Discussion

Scleral buckle infection is a prevalent postoperative complication of scleral buckling, with reported onset times categorized as early (two to eight weeks) or late (two months to four years), with this case presenting somewhere in between [3]. Regarding the pathogenesis of the infection, Chajablani et al. studied scleral buckle infection in 132 eyes [2]. They found that Gram-positive bacilli were the most common organisms, followed by Gram-negative rods, which together accounted for more than half of the cases. Pseudomonas aeruginosa was the most common Gram-negative rod, with Neisseria and Acinetobacter being the other isolates. *S. maltophilia*, which was detected in our case, is a Gram-negative rod, but it is rare as an initiator of scleral buckle infection and has only been reported in one case [4]. In that report, the infection was described as a combination infection with *Mycobacterium chelonae*, and it is unclear to what extent *S. maltophilia* contributed to the infection and inflammation.

Our patient continued on *S. maltophilia*-sensitive antibacterial medication, but the inflammation did not subside, resulting in intraocular inflammation and scleral thinning due to SINS. SINS is a rare inflammatory disease that occurs after ocular surgery. The time to onset of SINS varies widely from a few days to several decades after surgery, with an average duration of 5.7 months after surgery, and is more common in women than in men [5,6]. Doshi et al. reviewed the previous literature on the association between SINS and infection, examining postoperative scleral necrosis in 320 eyes, 203 after pterygium surgery, 56 after cataract/lens surgery, and 36 after scleral buckling [7]. The number of cases involving infection was 145 (71.4%) after pterygium, 16 (28.6%) after cataract and lens surgery, and 35 (97.2%) after scleral buckling, indicating that infection was more common in the pterygium and scleral buckle surgery. Of the 35 post-scleral buckling cases, Staphylococcus spp. were the most frequently detected organisms (18 cases), and *Pseudomonas aeruginosa* accounted for Gram-negative rods, but *S. maltophilia*, which was identified as SINS, was not included. SINS is also known to be associated with autoimmunity, but in the aforementioned article, 24 of 56 cases of SINS after cataract/lens surgery were found to have systemic pre-existing disease, and 14 were rheumatoid arthritis, while the frequency was low after scleral buckling (two of 36 eyes). The pre-existing bronchial asthma in this case might be a systemic immune-mediated disease. However, no reports on SINS and bronchial asthma have been found, and the association is unclear.

Some reports indicate improvement in SINS with oral non-steroidal anti-inflammatory drugs, however, long-term use is associated with side effects [5,8]. Therefore, oral steroids are recommended as the first-line treatment, with immunosuppressive drugs considered for refractory cases [6]. In our study, oral steroids were initiated at 30 mg/day, which demonstrated significant improvement, allowing for gradual dose reduction. Systemic steroid therapy was necessary despite concerns about exacerbating infection with steroids, emphasizing its importance in SINS treatment.

## Conclusions

Our patient developed a scleral buckle infection caused by *S. maltophilia* after scleral buckling for rhegmatogenous retinal detachment. This case is unusual because scleral buckle infection with *S. maltophilia* has been reported only once, and there has been no reported case of SINS. Treatment with antimicrobial agents alone was insufficient, necessitating the use of oral glucocorticoids as an anti-inflammatory agent.
